# Can Functional Performance Predict Long-Term Mortality Post Hip Fracture in Older Adults (65–100 Years of Age)?

**DOI:** 10.1093/geroni/igaa057.857

**Published:** 2020-12-16

**Authors:** Rashmita Bajracharya, Denise Orwig, Jay Magaziner, Jack M Guralnik

**Affiliations:** 1 University of Maryland, School of Medicine, Catonsville, Maryland, United States; 2 University of Maryland School of Medicine, Baltimore, Maryland, United States

## Abstract

Functional performance measures (grip strength, Short Physical Performance Battery (SPPB), and 3-meter gait speed) represent underlying disease progression and predict mortality. However, there is little information regarding whether these measures assessed at 2-months post-hip fracture predict long-term mortality (10-year follow-up). To address this gap, a longitudinal analysis of Baltimore Hip Studies-7 cohort, with mortality verified by National Death Index, was conducted. Mean difference in 2-month functional performance measures (n=242, men n=121, female n=121) among those who survived and did not survive over 10 years was determined using t-test. Prediction of mortality by these measures, overall and by sex, was estimated using cox proportional hazard models, for which Hazard ratios (HR) with 95% confidence intervals (CI) were estimated. We found that, gait speed [0.47(standard deviation,SD=0.39) versus 0.31(SD=0.27)] and SPPB score [4.89(SD=3.31) versus 2.83(SD=2.24)] were significantly higher at 2 months among those surviving compared to those who did not. Adjusting for covariates, functional performance predicted long-term mortality in men and women. Increase in gait speed by 0.1m/s predicted 15% decrease in mortality for men [HR=0.85(0.55-0.96)] and 17% for women [HR=0.83 (0.74-0.93)]. Increase in SPPB by 1 unit predicted decrease in mortality by 14% for men [HR=0.86(0.77-0.95)] and 17% for women [HR=0.83(0.74-0.93). Increase in grip strength by 1 kg predicted 5% decrease in mortality for men [HR=0.94(0.92-0.97)] and 9% for women [HR=0.90(0.86-0.95)]. Functional performance measured at 2-months post-hip fracture predicted long-term mortality. Those with poor functional performance at 2-months can be referred for further assessment to optimize their care to promote survival.

